# Medical and Philosophical Intelligence

**Published:** 1816-06

**Authors:** 


### MEDICAL AND PHILOSOPHICAL INTELLIGENCE.

*ROYAL SOCIETY of EDINBURGH.*—An account of the sleeping woman of Dunninald, near Montrose, was read by the Rev. James Brewster. Her first sleeping fit lasted from the 27th to the 30th of June, 1815. Next morning she again fell into a sleep which lasted seven days—without motion, food, or evacuation. At the end of this time, by moving her hand and pointing to her mouth, it was understood she wanted food, which was given to her; but she remained in her lethargic state till the 8th of August, —six weeks in all, without appearing to be awake, except on the 30th of June. Her pulse for the first two weeks was about 50; the third week 60; and previous to recovery it was at 70 to 72. Though extremely reduced, she gained strength so rapidly that before the end of August she worked regularly at the harvest. This case is well authenticated.

*The Medical Benevolent Society.*—The directors of this truly liberal and philanthropic institution, which embraces the interests of every medical practitioner resident in the united kingdom, who is a member of it, having, with the best legal assistance, finally arranged the laws and regulations by which it is to be governed; the society is, therefore, permanently established. Many liberal donations and numerous subscriptions have been received, forming in the aggregate a considerable sum of money, which has been invested in the public funds, conformably with the purposes expressed in the prospectus.

The particulars of the society may be known by application to Mr. Best, the Secretary, No. 15, Tavistock-street, Covent-garden.

In a former number we made mention of the unhappy differences concerning the medical officers at Bethlem. All the officers are annually annually elected as at the other royal hospitals; but, by custom, the re-election has been hitherto a mere matter of form. On the late occasion, the election of the physician and apothecary was suspended for a month, whilst the other officers, including the surgeon, were all continued. The general opinion of all impartial thinkers was, that this step was only an easy way of permitting the two former to resign. But these gentlemen either thought differently, or conceived that they had interest enough to maintain their places. On a show of hands, they were most pointedly, to use no severer term, disappointed. The apothecary, whose abilities are so well known by his publication was in a marked minority.

After this, the general meeting made a very prudent determination to have two physicians. Had they determined on three, we conceive it would not have been too many: two, however, is an important improvement in their establishment. The candidates for the offices are more numerous than we could have expected; we see, among the number, the son of the *ci-divant* physician. Should he succeed, it will make, we believe, the fourth in regular succession of the same family, three generations at least having already filled the office; the last held it for thirty-three years.

We expect much from the late attention paid to madness by the public; for, whatever may have been the researches of the faculty concerning that unhappy disease, Mr. Haslam's performance is the only one which offers any insight into the mode of treatment. The metaphysical reveries of Batty are not worth notice, and the answer by Monro is more a specimen of wit (said to be Smollet's), than calculated to afford information. The French writers have done something, but at present the pathology of madness is more in its infancy than any other disease. We forgot to mention Dr. Crighton's work, and probably most of our readers have forgotten that he ever wrote on *mental alienation*. In the want of more materials, it is, however, well worth consulting.

We trust the example of having two physicians will be followed by the governors of St. Luke's Hospital.

---

Mr. Brooke, of Keppel-street, has introduced a very valuable improvement in the Blow-pipe. When this is supplied by the mouth, the jette is always uncertain, and must be interrupted by the necessity the operator feels for respiration, besides which, he can only use one kind of air, the properties of which are somewhat uncertain, as there is always a mixture of pure atmospheric air with that which has been respired, and is forced from the lungs. The following account is extracted from Thomson's Annals of Philosophy.

"The occasion of my having this blow-pipe made, was to relieve the great inconvenience I felt in using the common blow-pipe with the mouth. The first idea that suggested itself to me was to produce the jet of air from a sort of artificial mouth, or *moveable* receiver, of rather large dimensions, the capacity of which should be capable of gradual reduction by means of a spring; but it im-

 mediately mediately occurred to me that the elasticity of the air itself, if forced into a *fixed* receiver, would be more uniform in its action than any spring, and might be regulated so as to produce a continued and more uniform jet. I accordingly applied to Mr. Newman, in Lisle-street, to make one upon this principle, to consist of a copper or iron vessel, into which the air is forced by a small condensing syringe, and from which it is suffered to escape through a tube of very small aperture, regulated by a stop-cock; and I have found it capable of affording a very intense and regular degree of heat. The form given in the drawing has been adopted by Mr. Newman for the convenience of packing into a small case; and he has also added to the syringe a screw, by means of which the receiver may be filled with oxygen or any other gas, which renders it more extensive in its application to chemical purposes, and probably so as to supersede the use of the common gazometer."

---

We are glad to find the following history has at length found its way, as an authentic record, in the Transactions of the Linnæan Society. The fact has hitherto only been noticed in fugitive periodical papers of the day.

" A hog was buried in its stye by a fall of part of the chalk cliff under Dover Castle, December 14, 1810. On the 23d of May, or 160 days after the accident, Mr. Mantell was told that some of the workmen employed in removing the fallen chalk had heard the whining of a pig. He encouraged them, in consequence, to clear away the chalk from the stye under the direction of the owner, Mr. Poole, who was present. He was soon afterwards surprised to see the pig alive extricated from its confinement. Its figure was extremely emaciated, having scarcely any muscles discernible; and its bristles were erect, though not stiff, but soft, clean, and white. The animal was lively, walked well, and took food eagerly. At the time of the accident it was fat, and supposed to have weighed about 160lbs.; but it now weighed only 40lbs. Mr. Mantell was assured that at the time of the fall there was neither food nor water in the stye, which is a cave about six feet square, dug in the rock, and boarded in the front; and the whole was covered about 30 feet deep in the fallen chalk. The door and other wood in front of the stye had been much nibbled, and the sides of the cave were very smooth, having apparently been constantly licked for obtaining the moisture exuding through the rock. There was no doubt that some of the loose chalk in front had been eaten; and from the appearance of the excrement, it may be conjectured that it had passed more than once through the intestines."

The above account is given by Thomas Mantell, Esq. F.L.S. Mayor of Dover, and Agent of the Packets at that place. An objection has been started that the creature was deprived of air. This, to us, appears more captious than philosophical. It is well known that in high northern latitudes bears are torpid during the winter; that animals, during that state, either cease to breathe, or respire so rarely that a small quantity of air is sufficient. This is particularly particularly remarkable in gregarious insects, who are clustered in such a manner as to exclude all air, and who emit, at those times, an air which will not support flame nor respiration. It will be found, on examining our Meteorological Register, that, at the period when the hog was first inclosed, the atmospheric temperature was higher than the freezing point, which may account for the sensation of hunger continuing till the air was so loaded with azote as to render the creature torpid, and induce its native habit of hybernating.

---

By Humboldt's account, published in the Prolegomena to his Nova Genera et Species Plantarum, the ancient Greek, Roman, and Arabian writers were not sufficiently acquainted with more than 1,400 plants, so as to describe and name them. At this time he enumerates 38,000 in the various quarters of the globe, viz.

| | |
|---|---|
| In Europe | 7,000 |
| Temperate regions of Asia | 1,500 |
| Asia, within the tropics and islands | 4,500 |
| Africa | 3,000 |
| Both temperate regions of America | 4,000 |
| America, between the tropics | 13,000 |
| New Holland, and islands in the Pacific | 5,000 |
| | 38,000 |

---

*Syphilis.*—It has been questioned by many whether it be possible to affect the system of a nursing child by quicksilver, through the medium of the mother. The following case proves the affirmative.

In the year 1808 a woman, who had an infant about six weeks or two months old at the breast, received the venereal disease from her husband. Being ignorant of the nature of her complaint, and trusting to common applications for a cure, it was allowed to proceed for some months before she applied to the writer of this article. The disease was found by him to exist locally to a great extent, accompanied with ulcers at the corners of the mouth, and on the nipples and breast. The matter of these ulcers coming into contact with the child had produced the same disease in and about its mouth, neck, and breast, to a great degree. In fact, the parent and offspring together presented a spectacle equally pitiable and disgusting. The child was ordered to be kept at the breast, and the mother was treated in the usual way by frictions, pills of sub-muriate of quicksilver, &c. A moderate salivation ensued, during which the face of the infant became swelled; a profuse discharge of saliva followed; the sores gradually healed; and they were both cured at the same time.—*New-Eng. Journ.*

The above case is interesting, inasmuch as it shows the progress of a morbid poison in a mother and child. The ulceration, *to a great degree*, is not like the secondary symptoms of syphilis, but like sivvens. We hope, from some succeeding numbers of that truly valuable journal, to learn a more minute description of the primary and secondary form of this disease.

Dr. Bayle,

Dr. Bayle, Physician to the King of France, and of the Hospital of Charity, has terminated a career, at the age of forty-two, which commenced in the most brilliant manner. Deprived of the external advantages for getting forward in the world, M. Bayle had no other resource than his talents. He was remarked, in his youth, as one of the best pupils of the School of Medicine in Paris; and he soon afterwards obtained the confidence of the public as rapidly as he had merited the esteem of his masters and fellow-students. Born in the Alps, M. Bayle preserved the habits of his early years: his exterior was far from prepossessing, his address displayed embarrassment and timidity; but a few moments of conversation dissipated these clouds, and displayed a mind comprehensive and solid, a refined understanding, a rare perspicuity, and a deep and various fund of scientific knowledge. The physician, the man of letters, the anatomist, and the natural philosopher, would have fancied that he had devoted himself exclusively to each of these branches of human knowledge.

M. Bayle had given himself up, for the last fifteen years, to the study of pathological anatomy. He published, a few years ago, Researches on Pulmonary Phthisis, founded on the dissection of upwards of 900 consumptive subjects. During his long illness, under which he fell, he put the finishing hand to a Treatise on Cancer; a work entirely new, and distinguishing, and carefully classing the various affections generally confounded under the name of Cancer. He wrote several articles in "the Dictionary of the Medical Sciences," and in various medical journals. He has left several scientific memoirs, some of which belong to the Society of the Faculty of Medicine, of which he was a member; and a vast number of notes, the result of his immense anatomical and medical researches.

M. Bayle united to great talents all the virtues which constitute the man of worth. Too skilful not to know long in advance the mortality of his disorder, he observed the progress, and foresaw the end with as much calmness as if it had been a patient. A few days before his death, he calculated even his last hour with a serenity of mind which that high sense of religion he had always displayed alone could give. The poor will long regret in him the physician who, in giving them advice, always enabled them to profit by it; his brethren, the modest man, who, even while young, was one of the luminaries of the French school; and his friends, a friend of tried probity, profound wisdom, and a sweetness and equality of temper, which was proof against all contingent events.

---

The following letter is transcribed from the last number of the Philosophical Magazine.

Sir,—In addition to what I have already communicated to you, in regard to my methods of preparing emetic tartar, I beg to submit to your readers a copy of a letter which I sent, above two years ago, to the president of the Royal College of Physicians, London.

 This

This I hope will serve as a protection against the officious and, I may say, illiberal, intrusion of one, who seems still determined to injure my professional reputation and, consequently, the welfare of my family.

I have said methods, because, by more ways than one, I have effected the decomposition, and have proved that an oxide of antimony, fit for the purpose, can be obtained directly from the common black sulphuret either by means of nitric acid alone, properly diluted with water, or the ingredients themselves by which this acid is prepared, together with the necessary quantity of water.

The process which I sent to you, and which you have already admitted into the Philosophical Magazine, has been supported by the most ample and favourable testimonies, both by manufacturers and others who are competent to judge of its merits; I have therefore no inducement at present to recommend any change in that prescription.

I take this occasion to declare that, excepting the preparation of the medicine in question, the tartarized antimony, I had less to do with the changes that have been made either in the former volume, or in the *editio nova* of the Pharmacopœia than may be supposed; I had no direct communication with the committee; nor do I know, even at this moment, how and of whom this committee was composed, whether it was open to all the members of the college, or secret and select; consequently I had no opportunity of preventing the material error (*tum cola*) which had unfortunately slipped into the formula for *antimonium tartarizatum.*

The letter, of which I shall subjoin a copy, was never meant to be kept as a secret, further than to be presented first to the consideration of the committee, which, as I understood, had been appointed to reform the Pharmacopœia of the London College. This letter, if admitted into your pages, will serve as a registry, notwithstanding all that has been asserted by one who has written so much and done so little, that the process which the college has now abandoned might have been perfectly modified; that the nitric acid was not in such great excess had a sufficient quantity of water been employed, and that the muriatic acid is not of so much consequence in the formation of protoxide of antimony, since the whole of the eleven fluid ounces there prescribed might have been omitted.

I remain, sir,

*Long-Acre;* Your obedient servant,

*April* 17, 1816. Jos. Hume.

*To Dr. Latham, &c. &c. &c.*

Sir,—A very simple and, I believe, effectual method of preparing tartarized antimony occurred to me yesterday, which I am anxious to present to you, lest I should be anticipated unfairly by any other experimentalist.

I shall not at present take up more of your time than to state the following sketch of the process.

The common black sulphuret of antimony is boiled with nitrous acid largely diluted with water. This produces an oxide of antimony mony which, after being properly washed, is to be boiled with supertartrate of potass and distilled water. The operation is then to be finished in the usual manner by evaporation and crystallization.

*Long-Acre;* I have the honor to be, sir, &c.
*Jan.* 21, 1814. Jos. Hume.

---

From our correspondent in Germany, we have received two cases of Hydrophobia cured by excessive bleeding, mercury, and other remedies; they will appear in our next.

---

Dr. John Reid is preparing a volume of Essays on Nervous and Hypochondriacal Diseases, and other Subjects.

---

Dr. Alexander Marcet will speedily publish an Essay on the Chemical History and Medical Treatment of Urinary Calculi, with plates.

---

Mr. Howship has nearly ready for publication some Practical Observations on the Diseases of the Urinary Organs, illustrated by Cases and Engravings.

---

Dr. Adam Dods has nearly ready for publication, the Physician's Practical Companion, as a Physico-Chirurgical Synopsis of Modern Medicine, arranged in alphabetical dissertations. Also, an Explanation of the Principal Technical Terms adopted by Ancient and Modern Writers.

---

Speedily will be published, some Practical Remarks in Surgery, illustrated by Cases. By A. Copland Hutchison, M. D. late Surgeon to the Royal Naval Hospital at Deal.

---

*Lectures on Anatomy.*—Mr. Carpue will commence his Anatomical Lectures at his Theatre, No. 50, Dean-street, Soho, on Monday, the 10th of June, 1816.

The Structure of the Human Body will be explained, as also its Physiology.

The Operation of Surgery will be shewn on the Dead Body.

Every day some part of the Human Body is demonstrated to the Pupils, who are required to demonstrate in their turn.—This plan obliges Mr. C. to limit the number of his Pupils.

Myology is not taught till the Pupils are perfectly instructed in Osteology, &c.

A general examination takes place every tenth day; and, if the Pupils do not perfectly recollect the parts that have been lectured on, they are again demonstrated.

The Dissecting-Room will be open from seven o'clock in the morning till five in the evening. The pupils are here taught the Method of operating, as also the Art of Injecting and making Preparations.

---

Dr. Squire and Dr. Davis will, about the middle of this month, begin a Course of Lectures on the Theory and Practice of Midwifery, and the Diseases of Women and Children.

### *Mr. Salisbury's Botanical Excursions and Calendar of Flora, for May* 1816.

Friday, May 3d, our excursion was to Battersea Fields, Wandsworth Common, and Tooting. The following few plants are all that were in bloom.

Cerastium vulgatum.
Juncus pilosus.
Montia fontana.
Saxifraga granulata.
Salix fragilis.
Ranunculus auricomus.
Salix caprea.
Fragaria sterilis.
Viola canina.
Viola odorata.
Primula vulgaris.
Viola tricolor.
Veronica serpyllifolia.
Saxifraga tridactylites.
Arabis thaliana.
Prunus spinosa.
Poa annua.
Lithospermum arvense.

Friday the 9th, a very wet day, and the excursion put off till Tuesday the 14th, when we visited Hampstead Heath, Cain Wood, and Highgate Archway.

Anemone nemorosa.

Pedicularis sylvestris.

Salix fusca.

Betula alba.

Prunus Cerassus.

Vaccinium Myrtillus.

Primula vulgaris.

——— officinalis.

Ranunculus bulbosus.

——— acris.

——— hirsutus.

Ajuga reptans.

Orobus tuberosus.

Plantago lanceolata.

Stellaria holostea.

——— graminea.

Lychnis diovica.

Acer. Pseudo-platanus.

Eryssimum Alliaria—just expanding the bloom.

——— Barbaria—ditto.

Veronica serpyllifolia.

——— arvensis.

Rumex Acetosella.

Geranium molle.

Scirpus sylvaticus.

Juncus sylvaticus.

Euphorbia sylvatica.

Scilla nutans.

Oxalis Acetosella.

Anthoxanthum odoratum. — This grass was not generally in bloom, the few specimens we found were growing on the warm bank at Highgate Archway.

The seeds of Tussilago farfara were this day quite perfect and flying about. The foliage of the Elms and some other trees not so forward as at Chelsea. Some of the Horse-chesnuts but just exfoliating.

May 17th. Charlton and Woolwich Sand-pits, and the Meadows opposite to Blackwall.

Myosotis scorpioides.

Draba muralis.

Medicago falcata.

Veronica Chamædrys.

Valeriana locusta.

Melica uniflora.

Orchis mascula.

Mercurialis perennis.

Fragaria vesca.

Pyrus Malus.

Arum maculatum.

Lycopsis arvensis.

Brassica Napus.

Brassica Rapa

Adoxa moschatellina.

Acer campestre.

Millium effusum.

Spartium scoparium.

Galeobdolon luteum.

Allium ursinum.

Cochlearia officinalis.

Geranium Robertianum.

Vicia Sepium.

Erodium cicutarium.

Alopecurus pratensis—only a few spikes to be seen in bloom.

Friday the 24th, Richmond Park, Combe Wood, and Wimbledon Common.

| | |
|---|---|
| Carex præcox. | Salix cineria. |
| ——— panicea. | Chærophyllum sylvestre. |
| Iberis nudicaulis. | Turritis glabra. |
| Ornithopus perpusillus. | Carex riparia. |
| Fagus sylvatica. | Hottonia palustris. |
| Ulmus campestris. | Genista anglica. |
| Poa nemoralis. | Sinapis arvensis. |
| Ilex aquifolium. | |

*General remarks.*—The grass in the meadows generally very luxuriant, and the fairest prospect of a heavy crop, though extremely late. The Horse-chesnuts in some places in full bloom, in others not a petal open. The foliage of the Hazel, the Elm, and the Hornbeam, nearly expanded to the full size. The Oaks in Richmond Park,* the foliage of which was so eaten up by the Cock-chafer last summer, have scarcely the buds broken; other Oak-trees in beautiful young leaf; but the stools of Oak in the low ground of Combe Wood, have not a single bud open, and are as perfectly naked as they were at Christmas. The Hawthorn is not in bloom that I have yet seen. The early variety of this shrub, called the Glastonbury-thorn, opened its blossoms on the 15th instant, and is now in the gardens in full bloom. Rye and winter tares—a large crop, and some are now cut for the first time. Wheat is in a fine healthy state, no appearance of disease, and in general a good regular crop. Clover fine, but very late. The spring-sown grain looks also very fine. The fruit on standards—Apples, Pears, Plums, and Cherries, are set in abundance, and are, in general, very promising, and at this late period they appear to have no other enemies than the insects. The Aphis lanata (*American blight*) on the Apple-trees, and the larva of the small red Beetle, mentioned at p. 90 of the work above quoted, are every day rapidly increasing.

The following days are intended for the excursions, and places of meeting in June:—

Friday the 7th, Battersea Fields and Wandsworth Common, Banks of the Thames, &c.

Friday the 14th, Regent's Park, Highgate Archway, and Hampstead Heath.

Friday the 21st, Woolwich Warren, and Chalk and Sand Pits at Charlton, in Kent.

Friday the 28th, Battersea Fields.†

At the desire of some of my students, I shall in future read a Lecture on Botany every Monday, at the Botanic Garden, at three o'clock. As some of your numerous readers may not understand my mode of teaching this science, and may wish to profit thereby, any persons of respectability will be admitted once gratuitously either to the lectures or excursions.

* Vide Observations on Orchards and on Insects that infest Fruit-trees, by W. Salisbury, lately published at the Botanic Garden, p. 73.

† Should it be wet weather on either of the above days, the excursion will be deferred till the following Tuesday, at the same place and time.

REPORT

### REPORT OF DISEASES.

Though inflammatory complaints still continue, they do not increase, either in number or violence. We have also met with several of those chronic diseases usually termed nervous. These are not among the number of such as have been copiously evacuated on account of previous inflammations; but, in some instances, we fear the effect of that despondency which the dull state of the metropolis induces among all classes—the artisans for want of employment, and the higher class of traders for want of remittances to answer their large consignments. Probably the fairer prospects which the improved condition of the funds, and the general tranquil state of Europe afford, may, with the cause, remove the effect. Inflammation, however, still exists sporadically in its most formidable shape, and requires the same energetic practice to arrest its progress. We have met with dysentery, which has pervaded a whole family, and proved fatal to a child two years old. This has been imputed to the emptying an old neglected sewer, and we are the more ready to admit such a cause, as the disease rarely occurs at this season, and as we have seen it in only this family. The symptoms were extremely violent in the fatal case, and not seen by the Reporter till ulceration had probably taken place over a considerable part of the mucous membrane of the intestines. The only other severe case was met with the lancet as soon as the *tormina* were considerable, and the patient is doing well.

The exanthemata continue general and severe. Small-pox, from the unaccountable supineness of the lower class of society, is gaining ground, though inoculation has almost ceased.

Colchicum has now, we conceive, established its reputation for the cure of gout. There are three preparations, all of which are kept secret, though the contents of each are pretty well known. The *eau medicinale* is supposed to be a strong infusion in some French wine. Wilson's (whose book we announced a few numbers ago, and which disappointed us in professing to keep the preparation a secret) is generally believed to be an infusion in cherry wine; and the last, invented for himself by a Bank clerk whose name we do not recollect, is said to be a strong watery decoction, with the addition of rum. Wilson's is most in use among the fashionable, who, we need not say, are the principal victims to the disease. The great expence of the *eau medicinale* has naturally suggested a most diligent attempt at its analysis; but its composition, like all other vegetable combinations, can only be guessed at by synthesis, and by the effects produced on the human constitution. Every thing of this kind confirms Mr. Want's opinion concerning colchicum, and lessens Mr. Moore's concerning helebore and opium.

### METEOROLOGICAL REGISTER.

### *From April the 26th, to May the 25th, 1816.*

Kept by C. BLUNT, Philosophical Instrument Maker, No. 38, Tavistock-Street, Covent-Garden.

| Moon. | Day. | Wind. | Barometrical Pressure. | | | Temperature. | | | |
|---|---|---|---|---|---|---|---|---|---|
| | | | Max. | Min. | Mean. | Max. | Min. | Mean. | |
| | 26 | SE | 30·13 | 30·12 | 30·125 | 69 | 49 | 59· | Fair |
| ● | 27 | SE | 30·10 | 30· | 30·05 | 67 | 48 | 57·5 | Fair |
| | 28 | SE | 30· | 30· | 30· | 64 | 37 | 50·5 | Rain |
| | 29 | SE | 30· | 29·97 | 29·985 | 66 | 39 | 52 5 | Rain |
| | 30 | SE | 29·84 | 29·58 | 29·71 | 65 | 38 | 51·5 | Rain |
| | 1 | SE | 29·74 | 29·68 | 29·71 | 64 | 38 | 51· | Rain |
| | 2 | SE | 29·89 | 29·82 | 29·855 | 63 | 36 | 49·5 | Rain |
| | 3 | SE | 30· | 29·96 | 29 98 | 61 | 39 | 50· | Rain |
| ◐ | 4 | SW | 30·11 | 30· | 30·055 | 62 | 40 | 51· | Rain |
| | 5 | W | 30· | 29 96 | 29·98 | 61 | 40 | 50·5 | Rain |
| | 6 | W | 29·96 | 29 93 | 29·945 | 60 | 38 | 48· | Rain |
| | 7 | W | 29·99 | 29·82 | 29·905 | 60 | 37 | 48·5 | Rain |
| | 8 | W | 29·74 | 29·68 | 29·71 | 59 | 36 | 47·5 | Rain |
| | 9 | W | 29·82 | 29·80 | 29 81 | 59 | 38 | 48·5 | Rain |
| | 10 | W | 29·50 | 29·42 | 29·46 | 57 | 35 | 46· | Rain |
| ○ | 11 | NE | 29·42 | 29 31 | 29·865 | 56 | 34 | 45· | Rain |
| | 12 | NE | 29·58 | 29·42 | 29·50 | 57 | 35 | 46· | Rain |
| | 13 | NW | 29·78 | 29 62 | 29.7 | 59 | 36 | 47·5 | Rain |
| | 14 | E | 29·90 | 29 89 | 29·895 | 58 | 37 | 47·5 | Rain |
| | 15 | SE | 29·90 | 29·89 | 29·895 | 56 | 41 | 48·5 | Fair |
| | 16 | SE | 29·93 | 29·83 | 29·88 | 66 | 40 | 53· | Fair |
| | 17 | E | 29·82 | 29 79 | 29·805 | 64 | 39 | 51·5 | Fair |
| | 18 | NE | 29·88 | 29·85 | 29 865 | 60 | 38 | 49· | Fair |
| ◑ | 19 | E | 29·88 | 29·85 | 29·865 | 60 | 40 | 50· | Fair |
| | 20 | SE | 29·90 | 29·90 | 29·9 | 59 | 38 | 48·5 | Fair |
| | 21 | SE | 29 97 | 29·97 | 29·97 | 58 | 34 | 46· | Fair |
| | 22 | E | 29·98 | 29·97 | 29·975 | 59 | 36 | 47·5 | Fair |
| | 23 | SE | 29·98 | 29·98 | 29 98 | 60 | 38 | 49· | Rain |
| | 24 | SW | 29·96 | 29·92 | 29·44 | 63 | 38 | 50·5 | Rain |
| | 25 | SW | 29·99 | 29·92 | 29·44 | 61 | 40 | 50·5 | Rain |

RESULTS.

| | | | |
|---|---|---|---|
| Mean barometrical pressure of the month | 29·8592 | Mean temperature of the month | 24·7452 deg. |
| Maximum 30·13, | wind at SE | Maximum 69, | wind at SE |
| Minimum 29·31, | ——— NE | Minimum 34, | ——— NE |

*Scale exhibiting the prevailing Winds during the Month.*

| N | NE | E | SE | S | SW | W | NW |
|---|---|---|---|---|---|---|---|
| 0 | 3 | 4 | 13 | 0 | 3 | 6 | 1 |

| | Mean barometrical pressure. | Mean temperature. |
|---|---|---|
| From the new moon on the 27th April, to the first quarter, on the 4th May | 29·898 | 51·785 |
| ——— the first quarter on the 4th, to the full moon on the 11th | 29·837 | 48·571 |
| ——— the full moon on the 11th, to last quarter on the 19th | 29·8 | 48·5 |

